# Supplementary material for: Infants’ Folate Markers and Postnatal Growth in the First 4 Months of Life in Relation to Breastmilk and Maternal Plasma Folate
Source: Nutrients. 2023 Mar 20;15(6):1495. doi: 10.3390/nu15061495 (PMC10051157; doi:10.3390/nu15061495)
Supplement: Supplementary file 1 [file nutrients-15-01495-s001.zip › nutrients-2227004-supplementary.pdf]

Table S1. Concentrations of folate status markers and catabolites measured in blood of exclusively breastfed infants and their mothers.

	Mothers	***Infants***		
	Visit 2 (8 weeks)[1]	Baseline (age < 1 month)[1]	Visit 4 (age 4 months)[1]	Change from baseline
Plasma 5-MTHF, nmol/L	20.0 (14.8)	26.1 (15.5)	34.3 (17.6)	7.7 (20.6)
Plasma pABG, nmol/L	7.4 (5.5)	8.5 (6.0)	14.8 (10.3)	6.5 (10.5)
Plasma N-acetyl-pABG, nmol/L	0.7 (0.3)	1.1 (0.4)	0.6 (0.3)	-0.5 (0.4)
Sum of pABG and N-acetyl-pABG, nmol/L	8.1 (5.6)	9.5 (6.0)	15.3 (10.3)	6.0 (10.5)
Sum of pABG and N-acetyl-pABG in nmol/L/kg infant body weight[2]	-	2.5 (1.6)	2.3 (1.5)	0.1 (2.0)
Plasma hmTHF, nmol/L	6.0 (3.1)	9.2 (6.3)	4.1 (3.0)	-5.1 (6.4)
Sum of 5-MTHF and hmTHF in plasma, nmol/L	26.0 (17.2)	35.1 (21.0)	38.3 (19.5)	2.8 (24.6)
Plasma tHcy, µmol/L	-	6.9 (2.1)	8.6 (2.8)	1.8 (1.7)
RBC-folate, nmol/L[3]	-	1387 (547)	1341 (605)	-67 (728)
Breastmilk 5-MTHF, nmol/L	46.3 (23.3)			

Data are shown as mean (SD).

[1] n = 114 infants at baseline; n = 112 infants at 4 months; and n = 113 women at visit 2 (8 weeks postpartum).

[2] The sum of the concentrations of pABG and N-acetyl-pABG at baseline visit and at visit 4 were divided by the individual body weights of the infants (in kg) at the corresponding study visits.

[3] The concentrations of RBC-folate were calculated from folate in whole blood hemolysate, plasma folate and hematocrit as described in the materials and methods session.

hmTHF, 4-alpha-hydroxy-5-methyltetrahydrofolate; 5-MTHF, (6S)-5-Methyltetrahydrofolate; N-acetyl-pABG, para-acetamidobenzoylglutamate; pABG, para-aminobenzoylglutamate; RBC, red blood cells; tHcy, total homocysteine.

Table S2. Concentrations of folate markers and catabolites in lactating women and their infants according to maternal plasma concentrations of 5-MTHF plus hmTHF dichotomized by the median.

Maternal folate markers (8 weeks postpartum)	Low maternal plasma concentrations of **5-MTHF plus hmTHF** (n = 56) Mean (SD) = 13.4 (4.2) Range 4.3-20.31 nmol/L[1]		High maternal plasma concentrations of **5-MTHF plus hmTHF** (n = 57) Mean (SD) = 38.3 (16.1) Range 20.32-109.7 nmol/L[1]		p^2 (low vs. high)		
Breastmilk 5-MTHF, nmol/L	49.3 (23.2)		42.4 (23.3)		0.054		
Plasma 5-MTHF, nmol/L	9.3 (3.5)		30.6 (13.9)		<0.001		
Plasma pABG, nmol/L	4.5 (2.7)		10.2 (6.0)		<0.001		
Plasma N-acetyl-pABG, nmol/L	0.6 (0.3)		0.8 (0.3)		<0.001		
pABG + N-acetyl-pABG, nmol/L	5.1 (2.7)		11.1 (6.1)		<0.001		
Plasma hmTHF, nmol/L	4.2 (1.9)		7.7 (2.9)		<0.001		
Infant's folate markers	Baseline	V4	Baseline	V4	p^3	p^4	p^5
Plasma 5-MTHF, nmol/L	24.1 (13.0)	35.3 (18.1)	27.3 (17.6)	32.4 (16.8)	0.538	0.288	0.284
Plasma pABG, nmol/L	7.5 (6.3)	14.6 (11.6)	9.1 (5.5)	14.6 (9.0)	0.015	0.759	0.869
Plasma N-acetyl-pABG, nmol/L	1.1 (0.4)	0.6 (0.3)	1.1 (0.3)	0.6 (0.2)	0.757	0.636	0.672
Plasma pABG + N-acetyl-pABG, nmol/L	8.5 (6.3)	15.3 (11.6)	10.3 (5.5)	15.2 (9.0)	0.017	0.737	0.904
pABG + N-acetyl-pABG nmol/L/kg body weight	2.2 (1.6)	2.3 (1.6)	2.7 (1.5)	2.4 (1.4)	0.018	0.739	0.903
Plasma hmTHF, nmol/L	8.2 (4.6)	4.3 (2.7)	9.8 (7.2)	3.8 (3.1)	0.327	0.160	0.166
Plasma tHcy, µmol/L	7.7 (2.3)	9.5 (3.3)	6.0 (1.3)	7.8 (2.0)	<0.001	0.573	0.511
RBC-folate, nmol/L	1147 (446)	1324 (639)	1628 (562)	1339 (567)	<0.001	0.262	0.328

Data are shown as mean (SD). Mean (SD) of infant's age was 20 (3) days at baseline and 113.4 (3.1) days at visit 4.

[1] Plasma concentrations of 5-MTHF plus hmTHF were dichotomized by the median value among the 113 participating women [median = 20.3 (min-max; 4.3-109.7) nmol/L].

[2] p values are from ANOVA test applied on the log_{10} transformed data.

[3] log_{10}-transformed concentrations at baseline visit were compared between strata of plasma folate in the mother by using ANCOVA test with age and weight of the infant at baseline visit.

[4] log_{10}-transformed concentrations at visit 4 were compared by using ANCOVA test with age and weight of the infant at visit 4 and the baseline concentrations of the same marker as covariates.

[5] ANCOVA test between concentrations at visit 4 was further adjusted for the MTHFR C677T and MTHFR A1298C genotypes of the infants.

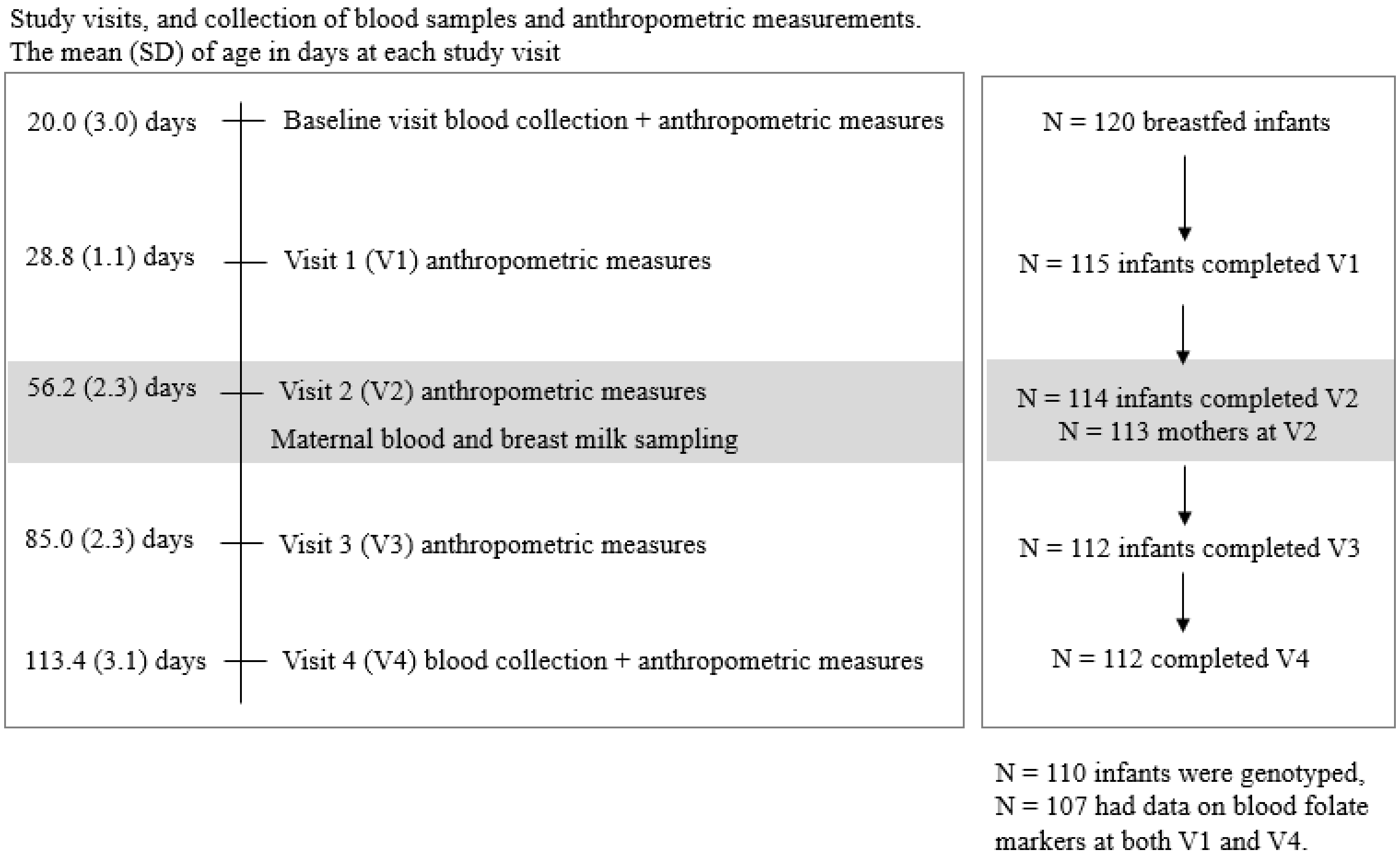

Figure S1. Study flow diagram.
